# Increasing myopia in Scotland at age of 3.5–5.5 years: A retrospective epidemiological study

**DOI:** 10.1111/opo.13461

**Published:** 2025-02-27

**Authors:** Bruce J. W. Evans, Lee Pentland, Benjamin E. W. Evans, David F. Edgar, Rakhee Shah, Miriam L. Conway

**Affiliations:** ^1^ Department of Optometry and Visual Sciences City St George's, University of London London UK; ^2^ Ninewells Hospital, NHS Tayside Dundee UK; ^3^ Hoya Vision Care Amsterdam the Netherlands

**Keywords:** children, myopia, school screening, Scotland, vision screening

## Abstract

**Purpose:**

Scotland has comprehensive child vision screening at age 3.5–5.5 years of age, with ~85% participation (40,000–50,000 screening episodes annually). Orthoptists deliver the screening, including presenting vision, cover test and other tests. Screening failures are referred for eye examinations, including cycloplegic refraction. The study aims to report refractive error data from these examinations (~5000–6000 annually) for 3 years before and 2 years following the COVID‐19 pandemic, and to investigate correlations between myopia and socio‐economic factors.

**Methods:**

Right eye data from eight Scottish health boards (HB) are reported for spherical equivalent refraction (SER) for the years 2013–14, 2014–15, 2015–16, 2020–21 and 2021–22. Associations were investigated between the proportion of the sample in each HB with myopia and the proportion of the population in each HB with different levels of deprivation index, classification on a rural/urban continuum and dwelling type.

**Results:**

Refractive error frequency distributions revealed a myopic shift in SER over the 5 years. Median SER (interquartile range) in 2013–14, 2014–15, 2015–16, 2020–21 and 2021–22 was +1.38 D (+0.50 to +2.75), +1.38 D (+0.25 to +2.63), +1.38 D (+0.50 to +2.75), +1.13 D (+0.25 to +2.25) and +1.25 D (+0.38 to +2.25), respectively. The increase in myopia was statistically significant in each of the last 2 years compared with each of the first 3 years (*p* < 0.0005). The proportion of myopes (≤−0.50 D) increased from <7.8% annually in 2013–16 to 11.51% in 2020–21 and 10.65% in 2021–22 (linear trend: *r* ^2^ = 0.94, *p* = 0.006). Associations between the proportion of children in each HB with myopia and: (1) deprivation index was low and not statistically significant; (2) the proportion of the population in the most urban environments was high (*r* ^2^ = 0.79; *p* = 0.003); (3) the proportion of dwellings that were flats/apartments was also high (*r* ^2^ = 0.83, *p* = 0.002).

**Conclusions:**

In this predominantly Caucasian population, the proportion of children with myopia has increased post‐COVID. A strong association exists between myopia and living in flats/apartments and urbanicity, but not with a deprivation index.

**Key points**

- In children aged 3.5–5.5 years, myopia has become more common in Scotland, United Kingdom. This may be a result of the COVID pandemic or other environmental/lifestyle changes among contemporary Scottish children.
- The increasing proportion of myopia detected by child vision screening raises questions about whether vision screening should be repeated periodically throughout the school years.
- Myopia is associated with living in flats/apartments and highly urbanised settings, but not with deprivation.

## INTRODUCTION

In Asian pre‐school^1^ and kindergarten^2^ children, myopia has emerged as more common than hypermetropia, affecting 22% of children by the age of 6 years (y).^3^ In Chinese children, Chen and colleagues reported that the age of myopia onset has steadily decreased over time, from a mean of 10.6 y in 2005 to 7.6 y in 2021 (*p* < 0.001).^4^

In the UK, McCullough et al. reported that, in 2016, for children aged 10–16 y, the proportion of myopes had more than doubled over the preceding 50 y and that children were becoming myopic at a younger age.^5^ Therefore, it is not surprising that an increase in myopia prevalence in young children is also evidenced in studies of Western populations.^6^, ^7^, ^8^, ^9^, ^10^, ^11^, ^12^, ^13^, ^14^ McCullough et al. noted that there were some differences between the methodology used in their cohort and the comparator data from Sorsby et al. in 1961.^5^, ^15^ Therefore, there is a need for further research on whether myopia is increasing in young children in the UK.

Before the COVID pandemic, there were signs that the prevalence of myopia in young Asian children, although high, had stabilised.^16^, ^17^ Several studies indicated that lockdowns, home schooling on computers^18^, ^19^ and/or less time outdoors^20^ as a result of the pandemic have been associated with an increased prevalence of myopia in children,^19^, ^21^, ^22^, ^23^, ^24^, ^25^, ^26^, ^27^, ^28^, ^29^, ^30^, ^31^, ^32^, ^33^ especially at young ages.^3^, ^34^, ^35^, ^36^ Most of these data came from Asian populations, but similar effects have been found in Spanish children aged 5–7 y^34^ and in Argentina.^28^ Klaver and colleagues described this increase in myopia as ‘quarantine myopia’ and noted that younger children may be particularly susceptible to environmental myopia triggers.^37^ Klaver et al. did not specify the environmental triggers,^37^ but these are summarised in a review by Biswas et al.^38^

Children's vision screening in the UK normally occurs at age 4–5 y with the primary goal of detecting amblyopia.^39^ More recently, a study^40^ noted that the UK National Screening Committee (NSC) recommendation^41^ is unusual in only screening children's vision once. A systematic review in 2021 argued that the UK vision screening programme is preferable for detecting amblyopia when compared with autorefraction or photorefraction at a younger age.^42^ Often, publications on vision screening still centre on the detection of amblyopia,^43^ although several studies have noted a much higher prevalence of refractive errors than amblyopia in preschool^1^ and school children.^6^, ^44^, ^45^ In children aged 4–5 y, previously undiagnosed visual defects are most likely to be refractive errors, and parents/carers are usually unaware of these.^46^ There is a higher risk of failing vision screening in families receiving benefits.^47^

McCullough and Saunders investigated child vision screening based on the UK NSC protocol in 294 children aged 4–5 y in Northern Ireland,^46^ and found moderately good sensitivity (70.4%) and specificity (82.2%), with the main difficulty being the detection of hypermetropia. No case of reduced vision as a result of myopia was undetected by screening.

The increasing prevalence of myopia has led to interest in vision screening for refractive errors,^8^, ^9^, ^48^, ^49^, ^50^ particularly myopia.^9^, ^51^, ^52^, ^53^ It is therefore not surprising that some authors have criticised the UK system of vision screening only at age 4–5 y, arguing for additional screening episodes at age 7 and 11 y,^54^ or one other screening intervention at age 11 y.^55^ A recent publication included a literature review of children's vision screening for myopia,^56^ and an analysis of data from over 300,000 computerised vision screening^57^ records from children in England aged 4–5 y. However, this report was confined to vision results, with no refractive error data.^56^

Since 2013, all children in Scotland who are registered with a General Medical Practitioner and are not already in the care of the Hospital Eye Service are invited to have vision screening as part of the See4School programme (https://www.nhsinform.scot/tests‐and‐treatments/routine‐tests‐and‐examinations/childrens‐vision‐screening/).^58^ Approximately 60,000 children, aged 3.5–5.5 y, are screened each year by orthoptists. Fully anonymised data are collated for basic audit purposes by the Scottish health boards and descriptions of these data have been published, including the overall performance of the screening programme.^58^, ^59^ In Scotland, cycloplegic refractive error data are obtained on those who fail the screening, and following referral attend the Hospital Eye Service or a community optometrist for an eye examination. The primary aim of this retrospective epidemiological study is to analyse these data to provide information on the proportions with myopia and the distribution of refractive errors in young children before and after the COVID pandemic.

At present, the influence of socio‐economic background on the risk of developing myopia or of having unmet visual needs owing to myopia is not fully understood.^60^, ^61^, ^62^ It is possible that the type of area (on a rural–urban continuum)^63^ or type of dwelling (e.g., detached, semi‐detached, terraced or flat/apartment)^64^, ^65^, ^66^, ^67^, ^68^ are more important factors in myopia development than socio‐economic factors. Additional secondary aims were to investigate whether the presence of myopia in young children in Scotland is associated with deprivation, urban (compared with rural) areas or flat/apartment dwellings (compared with other types of housing).

## METHODS

The research followed the tenets of the Declaration of Helsinki and proceeded after UK Health Research Authority and institutional approval and a data sharing agreement. The vision screening methods and pass/fail criteria are described by Pentland and Conway.^59^ The present analyses relate to children who, after failing screening, were referred to community optometrists or to the Hospital Eye Service. Optometrists or ophthalmologists carrying out the refraction were required to undertake cycloplegic refraction, using a method they considered appropriate. This is most likely to have been cycloplegic retinoscopy, but cycloplegic autorefraction and cycloplegic subjective refraction may also have been used when considered appropriate. Clinicians were requested to complete and return a form with refractive error data, which comprise the present dataset. In each year, the correlation between right and left eye spherical equivalent refraction (SER) was >0.85 and the right eye data are reported here (left eye data are included in Data S1). Myopia is defined as SER ≤ −0.50 D.^69^

Author LP, who is the Lead for Child Vision Screening in Tayside and co‐ordinates the audit of vision screening data in Scotland, merged data from different health boards and provided to the rest of the team deidentified data of screening and eye examination results in those who failed vision screening for the following years: 2013–14, 2014–15, 2015–16, 2020–21 and 2021–22. These years were selected because national data collection was started in 2013–14, and quality control checks were rigorously employed for the first 3 years and again from 2020‐2022. After data cleaning, data were checked independently by two co‐authors and any discrepancies resolved by discussion.

The National Health Service (NHS) Scotland Information Services Division (ISD) provided, for each year, the total number of children eligible for and invited to attend screening, the number who attended screening and the number who failed screening and were therefore referred for eye examinations. The ISD also provided information on the number of children wearing spectacles at screening.

### Statistical analysis

The refractive error data, which have not previously been reported, were analysed to address the primary aim using relative frequency distributions, descriptive and comparative statistics, proportion each year with myopia and regression of this proportion over time. Data were included for the eight health boards who provided a dataset for every year under analysis. For the relative frequency distributions, decisions about bin sizes, bin cut‐offs and to concentrate on right eye data were checked (see Results).

Some refractive error data were unavailable for analysis for two main reasons: either because the child failed to attend for an eye examination or because the clinician did not submit the form with the results of the examination. The missing data policy was to make no imputations for missing data (see limitations section of Discussion).

Typically, screening starts at the end of August (nursery term in Scotland starts in mid‐August) and ends in the summer of the following year, before the schools' return. In the year directly affected by the COVID pandemic (2020–21), screening started as usual in mid‐August 2020, but then there was a pause of approximately 2.5 months. To make up for the delay, many health boards set up screening clinics over the summer months (2021) in various locations (e.g., community centres, health centres and hospital clinics). In some health boards, the screening overran into the next screening year by up to 2 months (completion before the October 2021 mid‐term break). Therefore, it is likely that the mean age of screening would have been slightly older in the 2020–2021 year. The date of screening is not stored in the database available for analysis, and therefore, this delay cannot be quantified. This is considered further in the Discussion under limitations. For the 2021–22 year, the situation was back to normal.

The datasets available for analysis were fully anonymised: the only geographic data available for each participant were the health board in which they were screened. This facilitated analysis of the relationship between myopia rates in the present dataset and deprivation (ISD data), and from the Scottish Government official statistics (https://statistics.gov.scot/data), location (on an urban–rural continuum; urbanicity) and type of dwelling. These analyses were undertaken for the most recent year of refractive error data (2021–22). For deprivation, linear regression analyses were carried out to determine the relationship between the proportion of myopic children within each health board who failed vision screening and the proportion of children who failed vision screening and were within‐specific quintiles of deprivation for each health board. For urbanicity, the relationship between the proportion of cases of myopia who failed vision screening in each health board and the Scottish government population estimates on rural–urban classifications (2020) was investigated in each health board (https://www.gov.scot/publications/scottish‐government‐urban‐rural‐classification‐2020/documents/). For housing, the linear regression investigated was between the proportion of cases of myopia who failed vision screening in each health board and the most recent (2017) Scottish government data on dwelling type (https://statistics.gov.scot/data/dwellings‐type).

For each of the new variables (deprivation, urbanicity and housing), the analyses were restricted to two regressions for each hypothesis to avoid an excessive number of comparisons. These were for deprivation, the population within the lowest two quintiles (40%) and highest two quintiles (40%) of deprivation; for urbanicity, the percentage of the population in ‘large urban’ areas and those in rural areas (the rural category includes accessible rural, remote rural and very remote rural regions under the Scottish government's classification system) and for type of dwelling, those in flats/apartments and those in detached dwellings. To account for multiple comparisons, a Bonferroni correction was applied, so that for these analyses, results were considered statistically significant if *p* < 0.025 (i.e., 0.05/2).

## RESULTS

**FIGURE 1:**
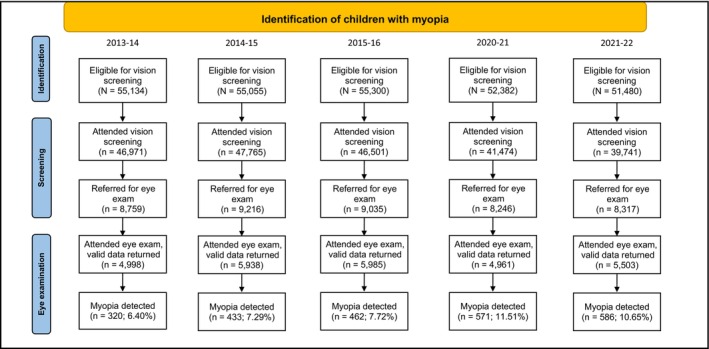
Preferred reporting items for systemic reviews and meta‐analyses (PRISMA) flow diagram summarising the identification of vision screening data used for the final analysis. For all years, the number who were eligible and attended for vision screening does not include children already under the Hospital Eye Service. See below for more details on samples at each stage.

The identification and screening stages in Figure 1 show data provided by the ISD for all health boards. The eye examination stage data are restricted to valid data returned by clinicians in the health boards included in the present analyses (see below). The number eligible for screening each year has fallen approximately in line with the falling birth rate (note that this appears exaggerated between the third (2015/16) and fourth years (2020/21) under study, but this was a 4‐year gap). Additionally, the number eligible for vision screening does not include children already under the Hospital Eye Service or for whom consent for screening was not provided.

The following eight health boards returned data for each of the 5 years investigated and are included in the eye examination data presented here, as summarised in the bottom two rows of Figure 1 (Ayrshire and Arran, Forth Valley, Greater Glasgow and Clyde, Grampian, Highland, Lanarkshire, Lothian and Tayside). The preferred reporting items for systemic reviews and meta‐analyses (PRISMA) chart in Figure 1 includes the number of children in each year analysed who were eligible and who attended for screening (children already under the Hospital Eye Service were not eligible for screening), were referred for an eye examination (using fail criteria detailed by Pentland and Conway),^59^ for whom refractive error data were returned by Hospital Eye Service clinicians or community optometrists and were found to have myopia in their right eye. Also shown is the proportion, each year, of individuals for whom valid eye examination data were returned who had myopia in their right eye.

Table 1 reveals a myopic shift in mean and median refractive error in the last 2 years of data (2020–22). A Kruskal–Wallis test indicated a statistically significant overall difference between years (*H* = 109.04, *p* < 0.0005), with pairwise comparisons showing statistically significant differences (*p* < 0.001) between each of the first 3 years and each of the last 2 years, but non‐significant differences in pairwise comparisons of each of the first 3 years (*p* > 0.22) and in a pairwise comparison of the last 2 years (*p* = 0.12). The proportion of children who failed screening criteria for presenting vision, subsequently attended for eye examination in the valid dataset and were myopic was <7.8% for each year from 2013 to 2016. This proportion increased to 11.51% in 2020–21 and 10.65% in 2021–22 (Figure 1 and Table 1, bottom row). There was a linear trend for an increasing proportion of children with myopia (*r* ^2^ = 0.94, *p* = 0.006).

**TABLE 1:** Descriptive statistics for frequency distributions of the right eye spherical equivalent refraction (SER) data for each year analysed.

Year	2013–14	2014–15	2015–16	2020–21	2021–22
*N*	4998	5938	5985	4961	5503
Mean (D) 95% CI of mean	+1.69 ±0.0016	+1.67 ±0.0016	+1.70 ±0.0016	+1.40 ±0.0017	+1.44 ±0.0016
Median (D)	+1.38	+1.38	+1.38	+1.13	+1.25
Std. Deviation (D)	+1.82	+1.93	+1.95	+1.94	+1.90
Minimum (D)	−11.75	−11.50	−11.50	−9.50	−16.00
Maximum (D)	+10.75	+12.50	+11.75	+9.88	+12.00
25th percentile (D)	+0.50	+0.25	+0.50	+0.25	+0.38
75th percentile (D)	+2.75	+2.63	+2.75	+2.25	+2.25
Number (%) with myopia	320 (6.40%)	433 (7.29%)	462 (7.72%)	571 (11.51%)	586 (10.65%)

Abbreviations: CI, confidence interval; D, Dioptre; N, number of refractive error records analysed; Std. Deviation, Standard deviation.

**FIGURE 2:**
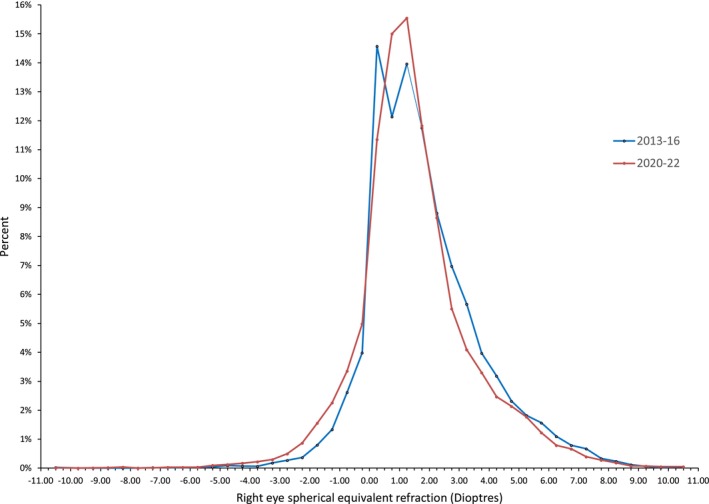
Graph showing the relative frequency distributions of right eye spherical equivalent refraction (SER) data for the first 3 years (blue) and the last 2 years (red) of data. Bin sizes are 0.50 D and the points plotted are the centre of each bin. Outlier bins have been collapsed so that the highest myopia bin (centred on −10.50 D) includes all cases who were <−10.00 D and the highest hypermetropia bin (centred on +10.50 D) includes all cases who were >+10.00 D.

For comparison of frequency distributions, since there were no statistically significant differences between the first 3 years (2013–14, 2014–15 and 2015–16), the data for these 3 years were combined and the blue line in Figure 2 shows the relative frequency distribution for these data. Similarly, since there was no statistically significant difference between the last 2 years (2020–21, 2021–22), the data for these 2 years were combined and the red line in Figure 2 shows the frequency distribution for these data. To facilitate comparison, the vertical axis represent the proportion (percentage) of the total sample for each period.

The relative frequency distributions (Figure 2) show a myopic (leftward) shift post‐COVID at virtually all refractive errors except the plano to +0.50 D bin, which had an unexpectedly high peak pre‐COVID. This is considered further in the Discussion.

The relative frequency distributions required several decisions that could have influenced the outcome (e.g., choice to use right eye data, decisions about bin sizes and cut‐offs and pooling the first 3 years and the last 2 years). These decisions and assumptions were checked by plotting frequency distributions for each year, each eye and using smaller bin sizes and different bin cut‐offs. These graphs and further analyses are presented in Data S1 and support the findings described above.

It is noteworthy from Figure 2 (and the graphs in Data [Link], [Link]) that the distribution of refractive error post‐COVID more closely resembles a normal distribution than the distribution pre‐COVID. This was investigated by comparing the distributions for 2013–16 and 2020–2022 with a normal distribution using the Kolmogorov–Smirnov test. The test statistic was higher post‐COVID than pre‐COVID (0.105 and 0.098, respectively). However, no doubt influenced by the large sample size, both frequency distributions differed significantly from a normal distribution (*p* < 0.001).

**FIGURE 3:**
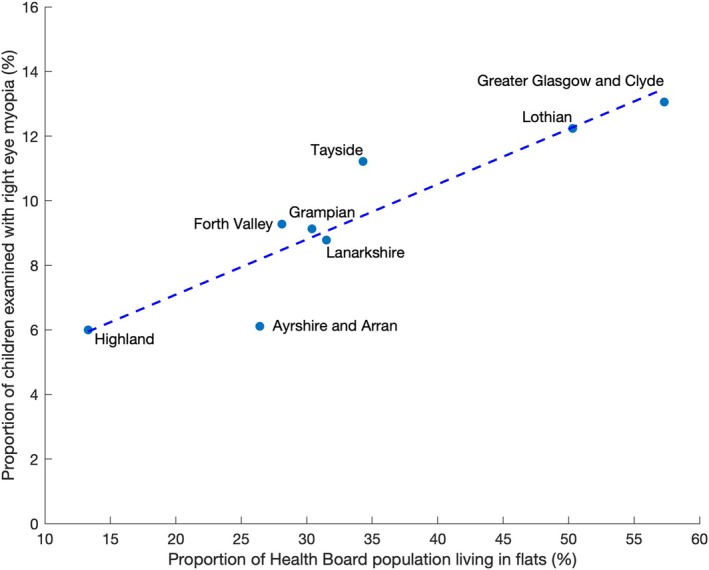
The relationship between the proportion of the population living in flats/apartments and the proportion with right eye spherical equivalent refraction (SER) ≤ −0.50 D within the eight Scottish health boards in the analysis. The dotted line is the linear regression (*r* ^2^ = 0.83, *p* = 0.002).

Regressions between the proportion of children in each health board who were myopes and the proportion of children who failed vision screening who fell within the lowest quintiles (1 and 2; most deprived) and the highest quintiles (4 and 5) of deprivation index were low and failed to reach statistical significance (*r* ^2^ < 0.07; *p* = 0.56 and *r* ^2^ < 0.01; *p* = 0.82, respectively). The strongest relationship was a high positive correlation (*r* ^2^ = 0.83, *p* = 0.002; Figure 3) between the proportion of children in each health board who were myopes and the proportion of dwellings that were flats/apartments. Conversely, there was a negative correlation that did not reach statistical significance between the proportion of children in each health board who were myopes and the proportion of dwellings that were detached houses (*r* ^2^ = 0.48, *p* = 0.06). There was a strong positive correlation between the proportion of individuals with myopia and the proportion of the population in the most urban environment (*r* ^2^ = 0.79, *p* = 0.003) and a non‐statistically significant negative correlation between the proportion of individuals with myopia and the proportion of the population living in rural environments (*r* ^2^ = 0.48, *p* = 0.06).

## DISCUSSION

Recently, reports on large databases of child vision screening data in England^56^ and Scotland^59^ described worsening presenting vision. The main finding of the new analyses reported here is that, in a large sample of children in Scotland aged 3.5–5.5 y, the proportion with myopia ≤−0.50 D has increased from 2013 to 2022 (from 6.40% to 7.72% pre‐COVID to 11.51% in 2020–21 and 10.65% in 2021–22). During the COVID pandemic, traditional schooling came to a halt and home learning became the new norm,^70^ with increased digital screen use^71^ and less daily physical activity.^70^, ^72^

It is well known that the frequency distribution of refractive errors approximates a normal distribution at birth but rapidly departs from this to form a leptokurtotic distribution, with low hypermetropia over‐represented.^73^ This is attributed to emmetropisation (although, semantically, ‘low‐hypermetropisation’ would be a more appropriate term). In the present data, it is noteworthy that the apparent evolution since the pandemic of the distribution of young children's refractive error is to more closely resemble a normal distribution (Figure 2). Flitcroft et al.^69^ considered that refractive errors that are present at 6 years of age can be considered as primary failures of emmetropisation, but added that the bulk of emmetropisation occurs in early childhood and is largely complete by 6 years of age. Certainly, the age of the children in the present cohorts is younger than the typical age of onset of school myopia.

Figure 2 shows a myopic (leftward) shift post‐COVID at virtually all refractive errors except the plano to +0.50 D bin, which had an unexpectedly high peak pre‐COVID. A possible explanation for this is that over the years analysed here, an increasing proportion of screening referrals were sent to community optometrists rather than to the Hospital Eye Service. The return rate of eye examination data from community optometrists was lower than from the Hospital Eye Service, and it is possible that community optometrists were less likely to return data when no spectacles were required. A related factor may be that children who failed the vision screening test with worse visual acuity in one or both eyes were more likely to be referred into the Hospital Eye Service than to community optometrists.

Vision screening in children has been found to be beneficial because it allows early detection and treatment of ocular anomalies^55^ and visual problems that may be missed in children.^57^ Uncorrected hypermetropia and myopia are linked to underachievement in educational assessments and poor academic performance, respectively.^74^

An attempt was made to reconcile the dataset described in this manuscript with data on eye examinations by community optometrists under the General Ophthalmic Services (GOS) in Scotland. However, such a comparison is prone to significant errors because of incompatibilities in the datasets (e.g., different age ranges and calendar years), and because a vision screening referral is only one of the reasons why children consult community optometrists. Also, when spectacle wearing children attended vision screening, they were screened while wearing their spectacles. This latter point may mean that the present work underestimates the proportion of children with myopia because some had already been corrected. This effect will be small because only 2%–3% of children each year wore spectacles to the screening appointments, and some of these would have had hypermetropia.

Other investigations support the present finding of an association between myopia and living in urban areas^63^ and small home size.^64^ There is uncertainty as to whether myopia is associated with socio‐economic status,^38^ and such a link was not evident in the present data. Individuals from socio‐economically disadvantaged backgrounds are prone to lower attendance rates at screenings and have higher failure rates in screening due to an increased prevalence of conditions including hypermetropia, esotropia and amblyopia.^75^, ^76^ Ethnicity, parental income, parents' level of education and attitude towards diagnosis and treatment may influence attendance at appointments.^77^ To be included in the dataset analysed here, families had to attend both pre‐school screening and eye care appointments with community optometrists or in the Hospital Eye Service, and any associations with lower socio‐economic status may therefore have been underestimated. Concerning eye examinations with community optometrists, Kearney et al. found that children with differing refractive errors living in deprived areas of Scotland were not disadvantaged in accessing NHS spectacles.^60^

Previous work has shown that both the type and size of housing are associated with myopia prevalence and progression rates.^64^, ^65^, ^66^, ^67^, ^68^ Housing can act as a proxy for socioeconomic and geographical conditions. The type and prevalence of housing, such as the relative number of flats/apartments, often correlates with both deprivation levels and urbanicity. Areas with a higher concentration of flats/apartments may indicate urban environments in addition to lower socioeconomic status due to their relative affordability and availability. A trivariate approach, utilising housing type in addition to deprivation indices and rurality, allows for a more holistic analysis of factors associated with myopia within this population. The results of the present analyses indicate that living in flats/apartments has the strongest association with myopia and urbanicity is less impactful, with deprivation far less relevant. A recent review noted that mid‐ to high‐spatial frequencies play an important role in the emmetropisation process and that there is a reduced high spatial frequency content indoors.^78^ This may be one reason why increased time outdoors reduces the risk of myopia. It is possible that children growing up in flats have less access to gardens, thereby reducing time outdoors.

Child vision screening in the UK has the primary goal of detecting amblyopia.^39^ In 2013, an external review against programme appraisal criteria asked whether the current UK screening at age 4–5 y met National Screening Committee (NSC) criteria.^79^ The review ‘found no robust evidence to support significant changes to the content of the current NSC recommended vision screening programme of children aged 4–5 y in the UK’, but did not consider whether a broadening of the programme was appropriate to include myopia and/or older children. In view of the increasing rates of myopia in the present data from children at age 3.5–5.5 y, it would be appropriate to re‐evaluate whether the emphasis of child vision screening should be to detect refractive error, including myopia, in addition to amblyopia.

Clearly, since 2016, a significantly elevated proportion of children in this dataset had myopia. For each year in the dataset, the proportion of children attending vision screening with spectacles was 2%–3%, and even whilst wearing their spectacles, most (63%–87%) of these children failed the vision screening, no doubt in some cases owing to progressing myopia. It seems that children with undetected or under‐corrected myopia cannot be relied upon to self‐refer. Older children may be more likely to self‐refer, but it is unsafe to assume that this will detect most cases of myopia.^46^, ^57^

In 2005, Logan and colleagues found that over 50% of university students in the UK were myopic.^80^ The rate of myopia post‐COVID in the present sample of approximately 10% applies to the population who fail vision screening, so the prevalence in the general population at age of 3.5–5.5 y is probably lower than 10%. Therefore, it seems likely that, throughout the school years, the prevalence of myopia increases from under 10% to probably over 50%, at least in those attending university. In a European population, Polling and colleagues showed that the median rate of progression of myopia is ~0.50 D per annum up to the age of 10 y and slower thereafter.^81^ Therefore, if vision screening is to be redesigned to target myopia as well as amblyopia, then in addition to early school vision screening, it will be necessary to repeat vision screening periodically during the school years.^54^, ^55^

It seems likely that the present findings are generalisable to other Western populations. It should be noted that the See4School programme involves vision screening by orthoptists, who are highly skilled in vision testing of young people. As explained by Pentland and Conway,^59^ the orthoptists had available three designs of logMAR letter chart tests so as to meet the needs of children with diverse abilities and also carried out additional orthoptic tests.

### Strengths and limitations

Strengths of the present work include the large sample size, which results in robust estimates of all the figures quoted in Table 1. Another strength is that the study population originated in community screening, rather than a clinical population who self‐refer to eye clinics.

A limitation of the present work is that, to avoid bias, data were restricted to those from health boards who returned vision screening results in every year analysed. Data from eight of the 14 health boards were analysed, and these eight were among the nine most populous in Scotland, comprising over 85% of Scotland's population according to the 2021 population data. Also, the gap in available data between 2016 and 2020 means that it is unclear whether the increase in the proportion of the sample with myopia is attributable to the COVID pandemic.

Another limitation is missing data: Refractive errors are only known for children who (i) attended and failed vision screening, (ii) were referred to community optometrists or the Hospital Eye Service, (iii) attended these clinics and (iv) for whom refractive error data were returned (Figure 1). No assumptions were made in the analyses regarding missing data. Data imputation^82^ was considered, but this requires assumptions that may be incorrect leading to erroneous conclusions.^83^ Not all children attend screening or follow‐up on referrals following screening, and the reasons for this were not explored in the present work. Of the children who were eligible for screening, the proportion who attended screening was ~85% in the first 3 years, which dropped to 79% and 77% in 2020–21 and 2021–22, respectively, which may have been related to the pandemic. This is unlikely to affect the main findings, because the rates of myopia were calculated by dividing the number of cases of myopia by the number who attended the eye examination, not the screening.

It is possible that children with symptoms are more likely to attend eye examinations after failing vision screening and this may have led to a slight overestimation of the proportion of children with myopia. However, few children at this age are likely to report symptoms, and there is no reason to believe that this possible source of bias would have changed over the years under study. Thus, the increasing myopia found in this investigation is likely to be a robust finding.

As a result of the delay in screening in the year of the pandemic (2020–21), the mean age of participants at the time of screening is likely to be older in that year than in other years. Since myopia becomes more common in children with increasing age, this is likely to contribute to the increased rate of myopia in this particular year. The date of screening and age were not stored in the database available for analysis, and therefore, this delay cannot be quantified. In Northern Ireland, McCullough et al. estimated the annual incidence of myopia at age 6–7 years to be 2.2%.^5^ Therefore, the delay of a few months for some participants in 2020–21 is unlikely to explain the increase in prevalence from under 8% pre‐COVID to 11.5% in 2020–21. Also, in 2021–22, when 10.65% of the cohort were myopic, the date of screening and therefore age at the time of screening are likely to have returned to levels similar to those in the pre‐COVID years. The distribution of refractive errors in 2020–21 did not differ significantly from that in 2021–22.

It should be noted that the correlational analyses to investigate the secondary aims break down the data by health board to look for broad trends. Given the large numbers, this is likely to be applicable and valid. However, a more granular analysis, where data for each child's dwelling type, postcode, level of deprivation, ethnicity, parental income, etc. were analysed, would eliminate the statistical assumptions inherent in the approach taken here. This was not possible due to data protection limitations. Additionally, the comparisons across health boards only assessed specific factors (deprivation quintiles, urbanicity and dwelling type) and it is possible that other factors could have been relevant.

In conclusion, in a large population of mainly Caucasian children in Scotland aged 3.5–5.5 y who have failed vision screening, myopia became more common from 2016 to 2022. Even at this young age, approximately one in 10 children in the post‐pandemic dataset who failed vision screening and were analysed here was myopic. This raises concerns about the need to repeat vision screening periodically during the school years. Myopia is particularly likely to affect those living in highly urbanised locations and in flats/apartments.

## AUTHOR CONTRIBUTIONS

**Bruce J. W. Evans:** Conceptualization (equal); data curation (supporting); formal analysis (equal); funding acquisition (lead); investigation (supporting); methodology (equal); project administration (equal); validation (equal); visualization (equal); writing – original draft (lead); writing – review and editing (equal). **Lee Pentland:** Conceptualization (equal); data curation (lead); formal analysis (equal); funding acquisition (supporting); investigation (lead); methodology (equal); project administration (equal); validation (equal); visualization (equal); writing – original draft (supporting); writing – review and editing (equal). **Benjamin E. W. Evans:** Conceptualization (equal); data curation (supporting); formal analysis (equal); funding acquisition (supporting); investigation (supporting); methodology (equal); project administration (equal); validation (equal); visualization (equal); writing – original draft (supporting); writing – review and editing (equal). **David F. Edgar:** Conceptualization (equal); data curation (supporting); formal analysis (equal); funding acquisition (supporting); investigation (supporting); methodology (equal); project administration (equal); validation (equal); visualization (equal); writing – original draft (supporting); writing – review and editing (equal). **Rakhee Shah:** Conceptualization (equal); data curation (supporting); formal analysis (equal); funding acquisition (lead); investigation (supporting); methodology (equal); project administration (equal); validation (equal); visualization (equal); writing – original draft (supporting); writing – review and editing (equal). **Miriam L. Conway:** Conceptualization (equal); data curation (lead); formal analysis (equal); funding acquisition (supporting); investigation (supporting); methodology (equal); project administration (equal); validation (equal); visualization (equal); writing – original draft (supporting); writing – review and editing (equal).

## CONFLICT OF INTEREST STATEMENT

The data analysis and manuscript preparation were funded by HOYA. Rakhee Shah is an employee of HOYA.

## Supporting information


Data S1.

Data S2.

## Data Availability

The full eye examination dataset will be made available following any reasonable request to the authors.
