# Supplementary material for: Increasing myopia in Scotland at age of 3.5–5.5 years: A retrospective epidemiological study
Source: Ophthalmic Physiol Opt. 2025 Feb 27;45(3):834–44. doi: 10.1111/opo.13461 (PMC11976510; doi:10.1111/opo.13461)
Supplement: Supplementary file 1 — Data S1. [file OPO-45-834-s002.pptx]

## Slide 1
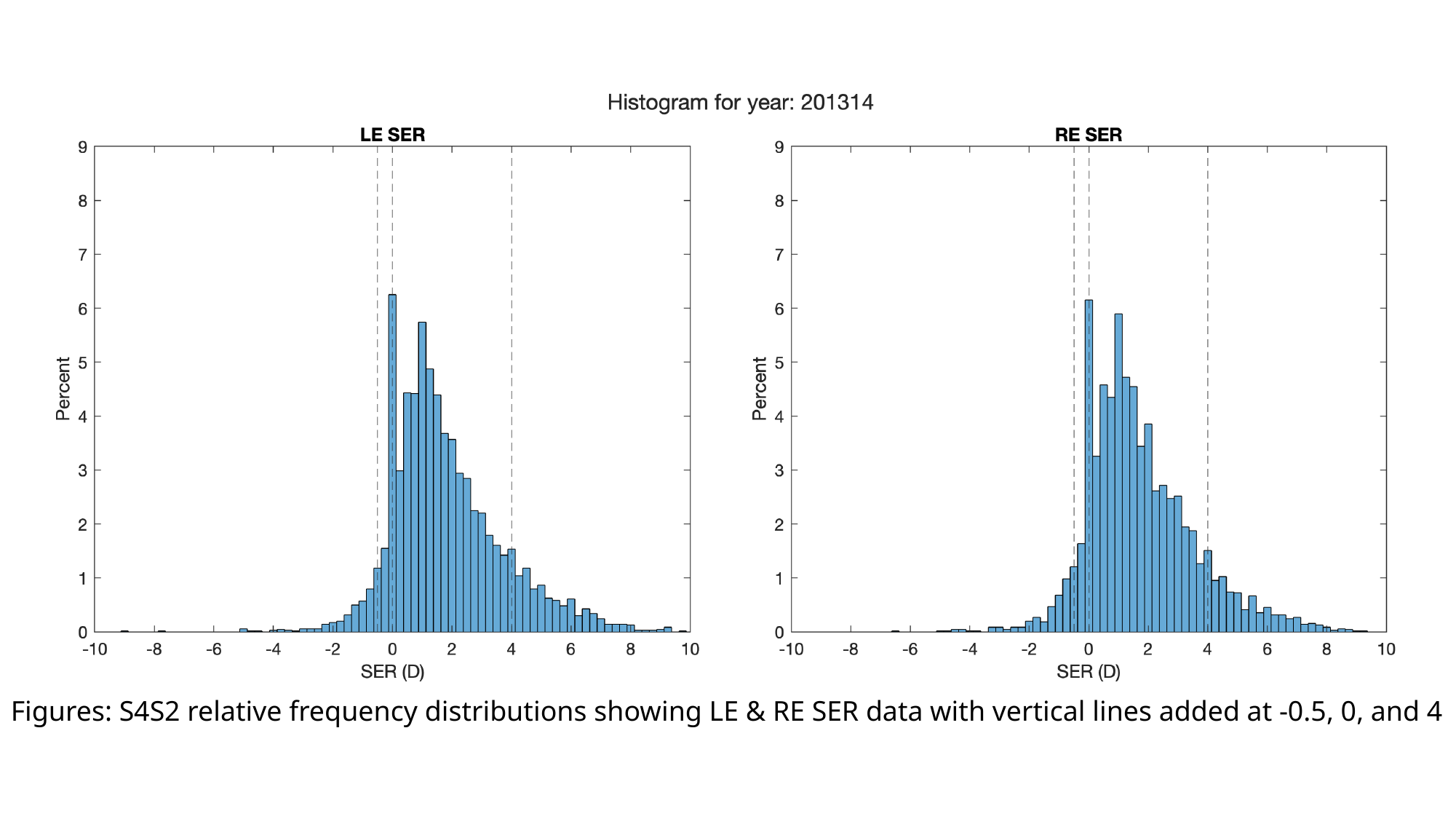

Figures: S4S2 relative frequency distributions showing LE & RE SER data with vertical lines added at -0.5, 0, and 4

## Slide 2
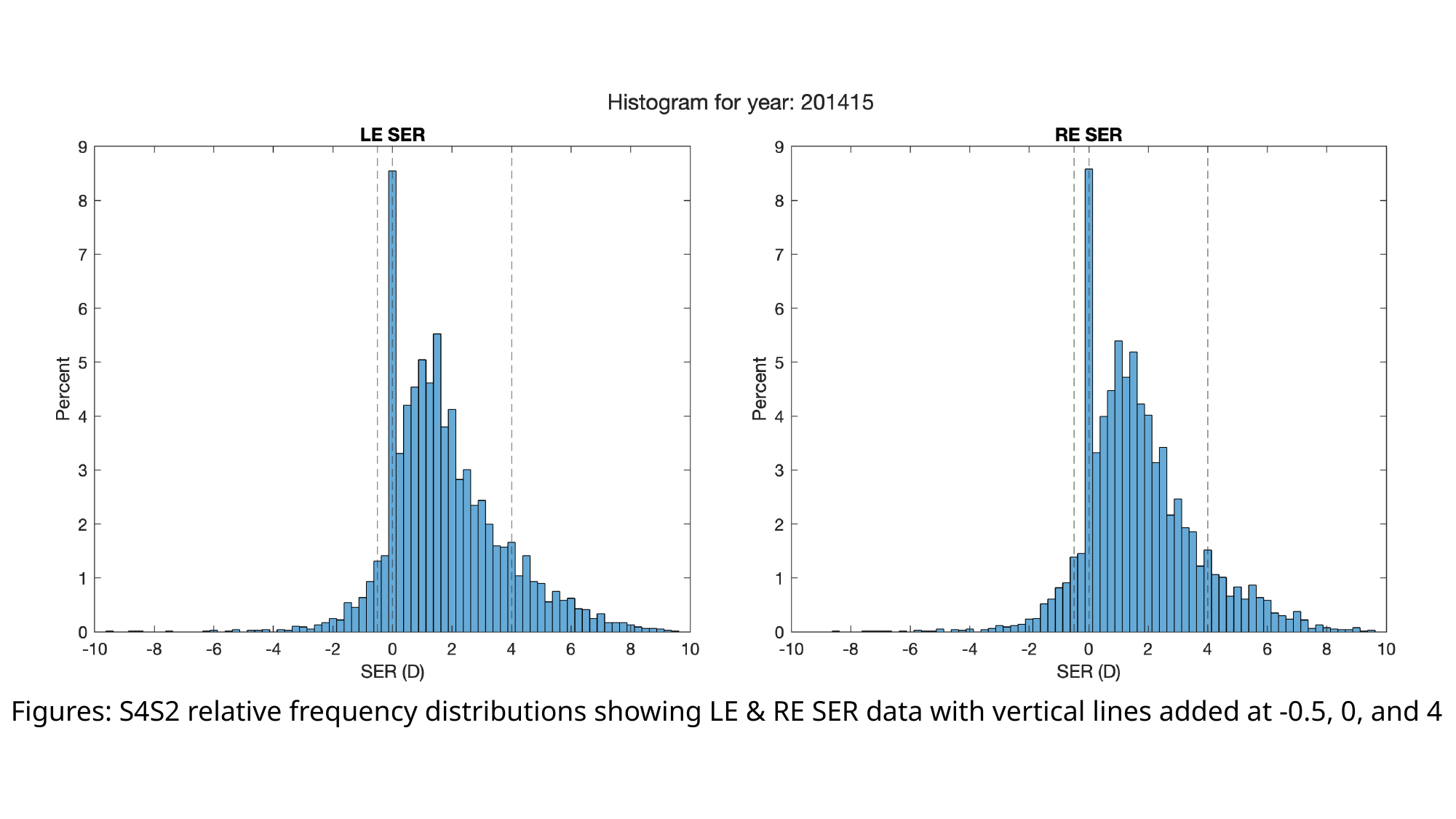

Figures: S4S2 relative frequency distributions showing LE & RE SER data with vertical lines added at -0.5, 0, and 4

## Slide 3
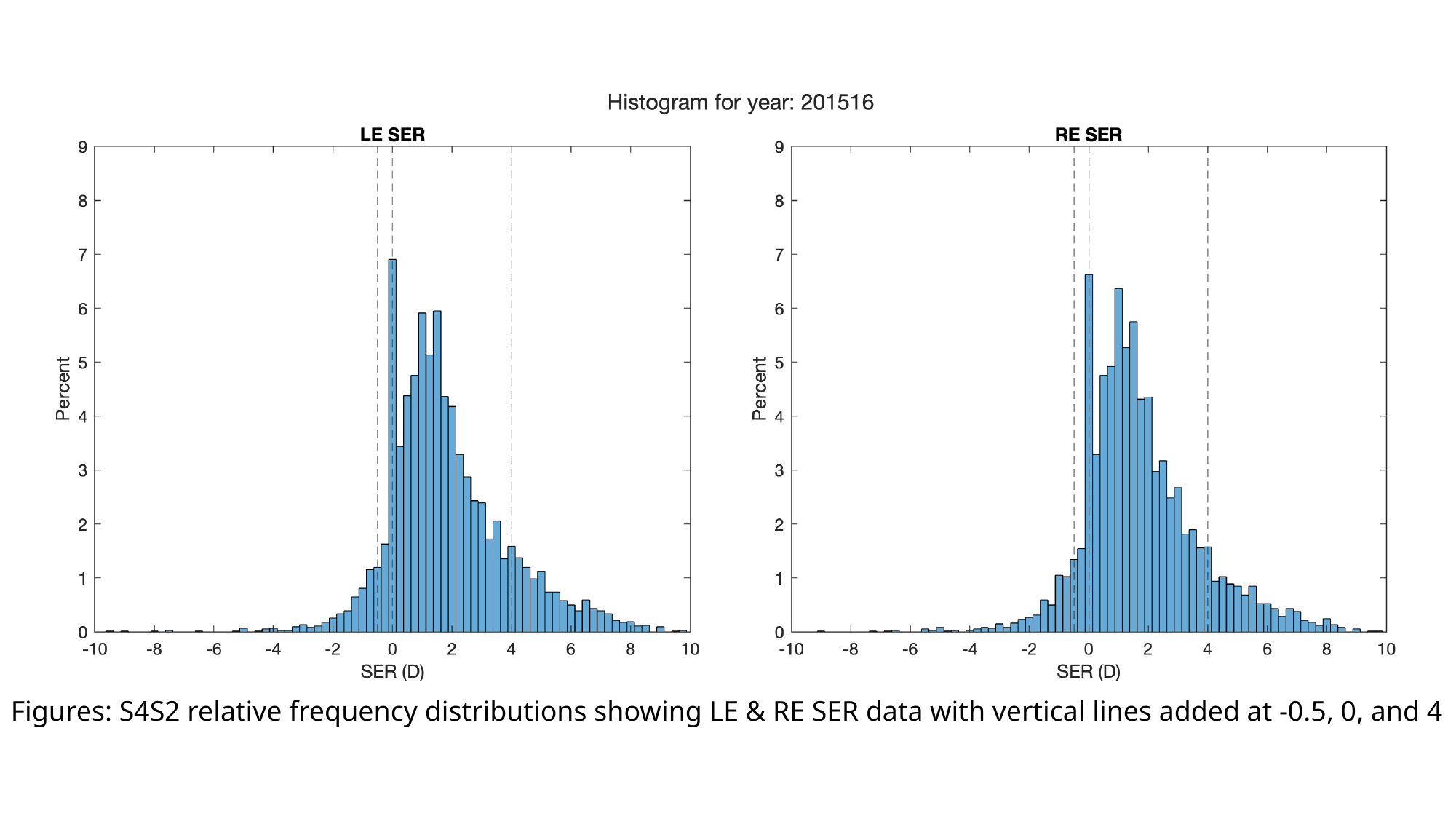

Figures: S4S2 relative frequency distributions showing LE & RE SER data with vertical lines added at -0.5, 0, and 4

## Slide 4
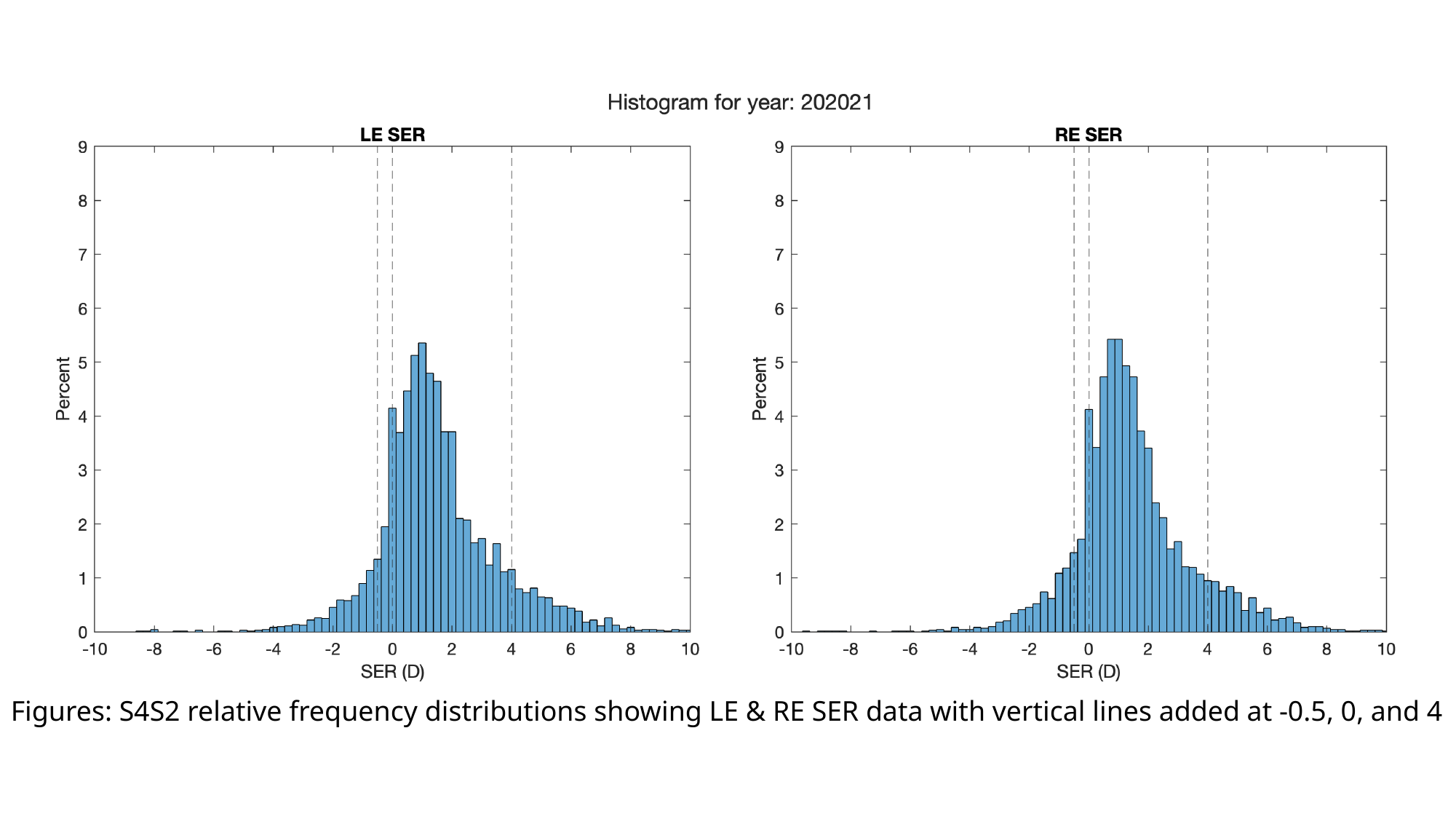

Figures: S4S2 relative frequency distributions showing LE & RE SER data with vertical lines added at -0.5, 0, and 4

## Slide 5
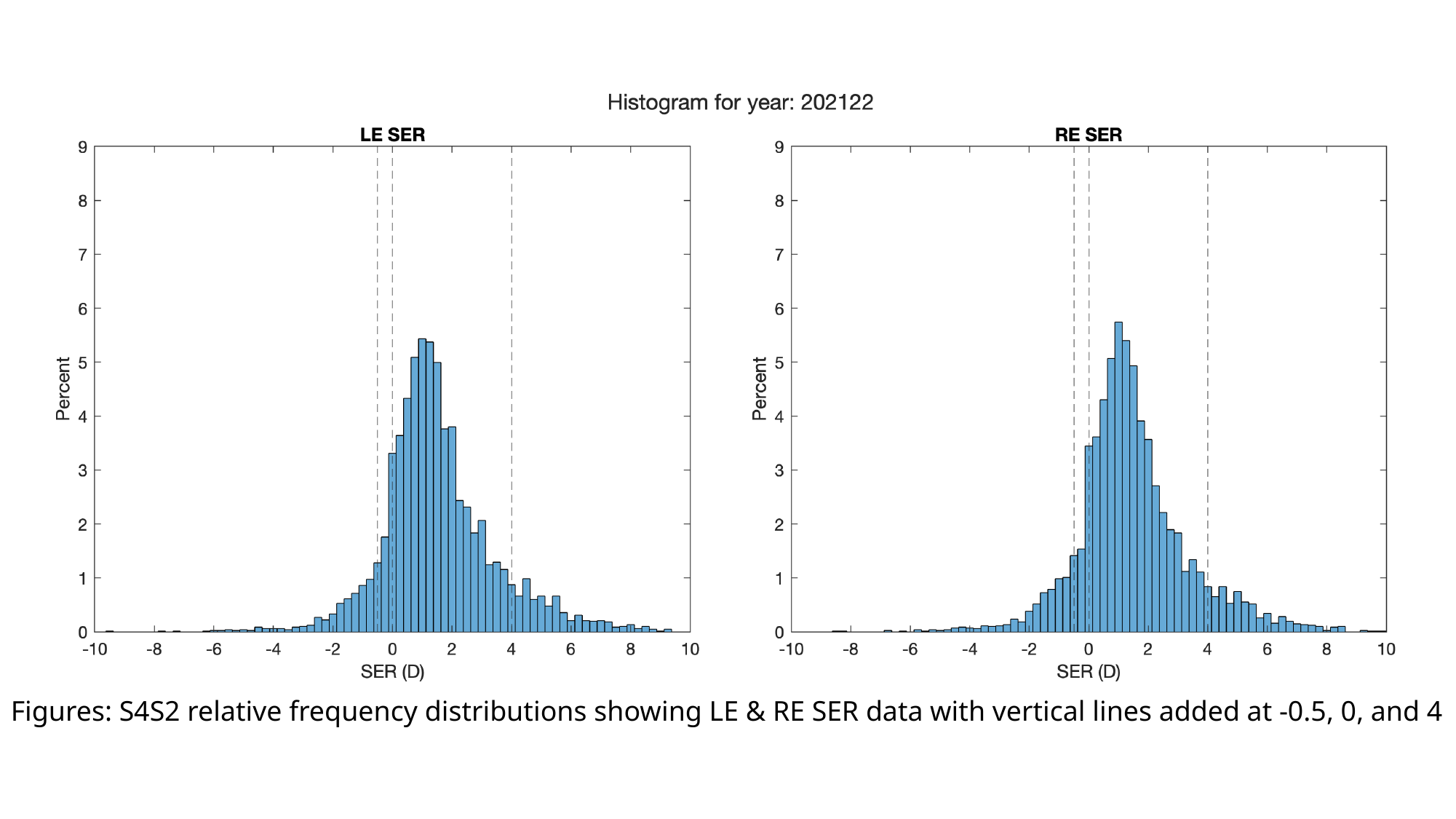

Figures: S4S2 relative frequency distributions showing LE & RE SER data with vertical lines added at -0.5, 0, and 4
