# Supplementary material for: Increasing myopia in Scotland at age of 3.5–5.5 years: A retrospective epidemiological study
Source: Ophthalmic Physiol Opt. 2025 Feb 27;45(3):834–44. doi: 10.1111/opo.13461 (PMC11976510; doi:10.1111/opo.13461)
Supplement: Supplementary file 2 — Data S2. [file OPO-45-834-s001.pptx]

## Slide 1
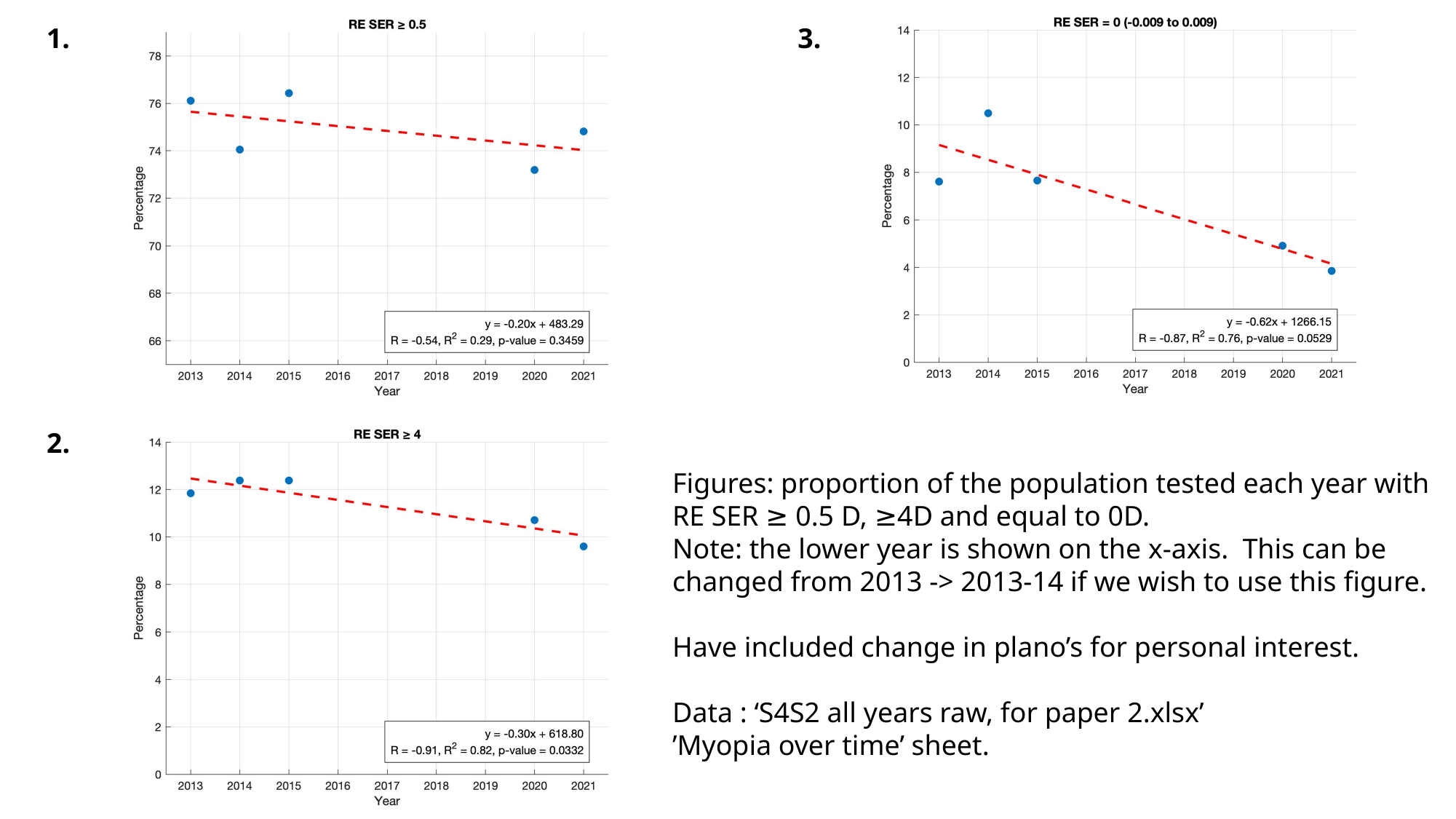

1.
3.
2.
Figures: proportion of the population tested each year with RE SER ≥ 0.5 D, ≥4D and equal to 0D.
Note: the lower year is shown on the x-axis. This can be changed from 2013 -> 2013-14 if we wish to use this figure.
Have included change in plano’s for personal interest.
Data : ‘S4S2 all years raw, for paper 2.xlsx’
’Myopia over time’ sheet.

## Slide 2
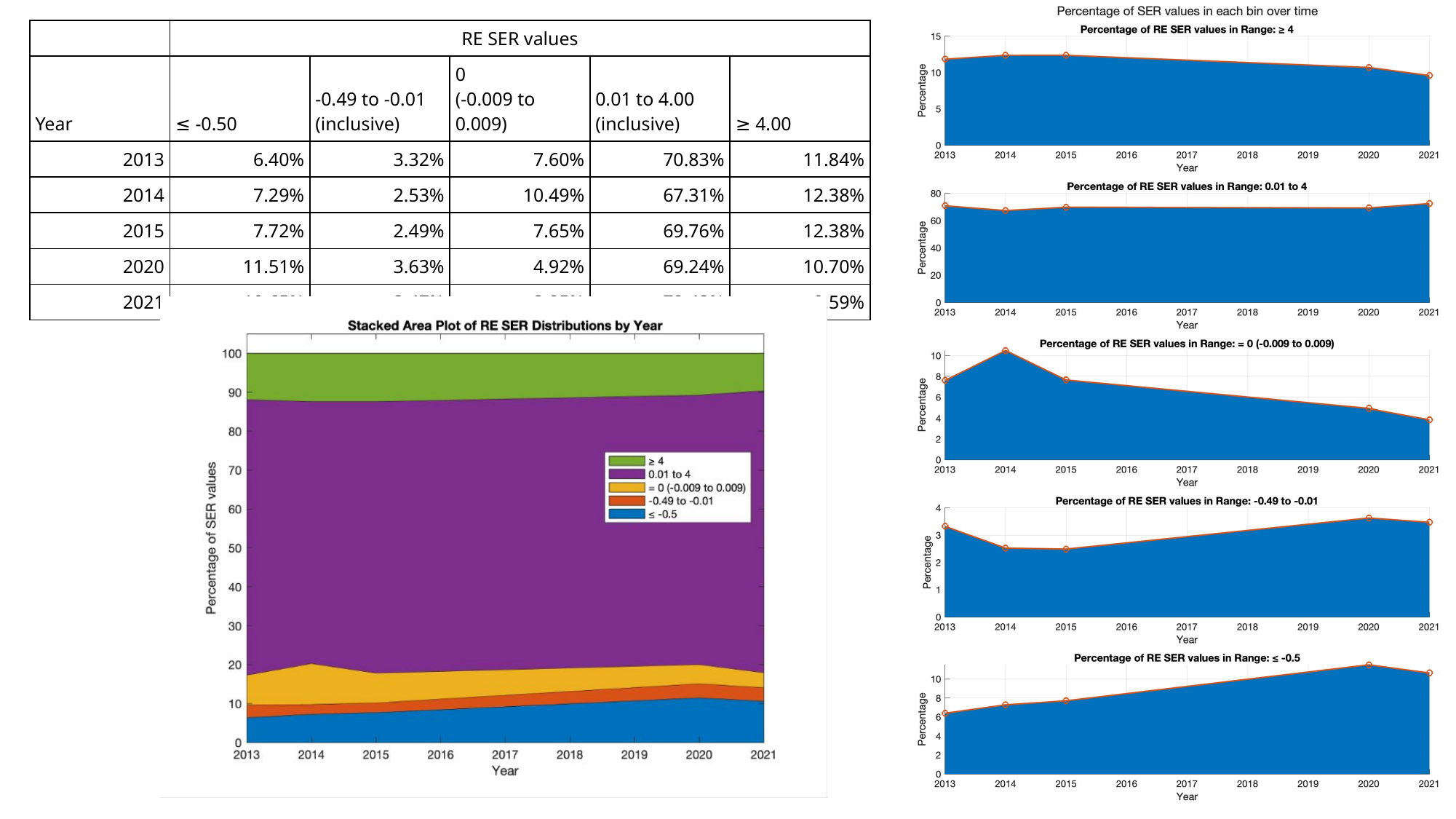

| | RE SER values | | | | |
| --- | --- | --- | --- | --- | --- |
| Year | ≤ -0.50 | -0.49 to -0.01 (inclusive) | 0 (-0.009 to 0.009) | 0.01 to 4.00 (inclusive) | ≥ 4.00 |
| 2013 | 6.40% | 3.32% | 7.60% | 70.83% | 11.84% |
| 2014 | 7.29% | 2.53% | 10.49% | 67.31% | 12.38% |
| 2015 | 7.72% | 2.49% | 7.65% | 69.76% | 12.38% |
| 2020 | 11.51% | 3.63% | 4.92% | 69.24% | 10.70% |
| 2021 | 10.65% | 3.47% | 3.85% | 72.43% | 9.59% |
